# Precision Delivery of Steroids as a Rescue Therapy for Gastrointestinal Graft-versus-Host Disease in Pediatric Stem Cell Transplant Recipients

**DOI:** 10.3390/jcm12134229

**Published:** 2023-06-23

**Authors:** Steven Levitte, Abantika Ganguly, Sophie Frolik, Alix A. Guevara-Tique, Shaini Patel, Ann Tadas, Orly Klein, David Shyr, Rajni Agarwal-Hashmi, Lynn Beach, Elizabeth Callard, Katja Weinacht, Alice Bertaina, Avnesh S. Thakor

**Affiliations:** 1 Interventional Radiology Innovation at Stanford (IRIS), 3155 Porter Drive, Palo Alto, CA 94304, USA; 2 Division of Pediatric Gastroenterology, Hepatology, and Nutrition, Stanford University, Palo Alto, CA 94304, USA; 3 Department of Pediatric Radiology, Interventional Radiology, Stanford University, Palo Alto, CA 94304, USA; 4 Division of Pediatric Hematology/Oncology, Stem Cell Transplantation, and Regenerative Medicine, Stanford University, Palo Alto, CA 94304, USA

**Keywords:** GVHD, stem cell transplant, intraarterial, intestinal GVHD, locoregional

## Abstract

Graft versus host disease (GVHD) is one of the most serious complications following stem cell transplant in children and is a major cause of morbidity and mortality. Corticosteroids remain the mainstay of treatment, and although a majority of children respond to systemic steroids, those refractory to or dependent upon corticosteroids suffer from complications secondary to long-term steroid administration. This problem has prompted consideration of steroid-sparing treatment strategies, although the time to clinical remission can be variable. Intraarterial corticosteroid delivery has been used in adults as a rescue therapy in steroid-resistant patients, but its use in children has been limited. We investigated the feasibility of intraarterial steroid administration into the bowel and/or liver in a cohort of six pediatric patients with acute GVHD. All patients successfully underwent treatment with no serious adverse effects. Five of five (100%) patients with gastrointestinal bleeding due to GVHD had rapid symptom improvement by 48 h, which was durable up to three weeks. Three of four (75%) patients with hepatic GVHD had improved cholestasis following intraarterial steroid administration. Our experience with this small cohort preliminarily demonstrated the feasibility and safety of intraarterial steroid administration in children with acute GVHD. This approach warrants consideration as a rescue therapy in steroid-refractory cases and as a “bridge” therapy for children with severe acute GVHD who are transitioning to steroid-sparing regimens.

## 1. Introduction

Graft-versus-host disease (GVHD) is a serious complication following allogenic stem cell transplantation, which can result in life-threatening inflammation and tissue damage affecting multiple organs including the liver, bowel, skin, and bone marrow. Acute GVHD remains the most feared complication of allogeneic stem cell transplantation (SCT) in children, affecting roughly 25% of HLA-matched transplants and a higher percentage of mismatched transplants [1]. Treatment generally includes systemic corticosteroids to reduce inflammation, although some children (~30%) develop steroid-refractory or steroid-dependent disease, which can become life-threatening [2]. Unfortunately, there are currently no biomarkers that can predict the likelihood of steroid responsiveness. As a result, many children who develop GVHD are treated with prolonged courses of high-dose steroids despite an array of complications including infections, diabetes, hypertension, muscle weakness, anxiety, and insomnia. In addition, steroids are associated with long-term health issues like bone damage, cataracts, and decreased linear growth [3]. These side effects make systemic steroids unsuitable for long-term therapy, especially in children. Moreover, there is no established standard second-line treatment for patients with steroid-resistant, or -dependent, disease.

Steroid-sparing therapy is becoming more common, particularly for bowel GVHD, in which biologics such as vedolizumab and ustekinumab are being increasingly used to control inflammation [4,5]. However, these therapies can take weeks to reach therapeutic levels, hampering their use in acute GVHD treatment [6]. Ruxolitinib has shown promise as a treatment for steroid-refractory acute GVHD following SCT, although the time to response can vary widely [7].

The intraarterial local delivery of corticosteroids is an innovative technique under investigation in multiple inflammatory conditions [8]. It not only allows for a higher dose to reach the precise affected area but also significantly reduces systemic side effects. Multiple studies have shown the potential for intraarterial steroids as a bridge therapy in adult GVHD, with only select case reports on children [9,10,11,12]. This is unfortunate because pediatric patients are ideal candidates for intraarterial therapies given the narrow therapeutic index that many drugs have in children and the relative safety of this intervention [13,14,15,16]. Here, we report our preliminary experience using intraarterial methylprednisolone for intestinal and/or hepatic acute severe GVHD in a cohort of six pediatric patients.

## 2. Materials and Methods

### 2.1. Human Subjects

Consecutive patients who developed GVHD of the liver and/or intestine between 1 January 2017 and 1 December 2022 and who underwent at least one procedure for administration of intraarterial methylprednisolone at a pediatric academic referral center were included in this analysis. This study was conducted with Institutional Review Board approval (#68632). There were no exclusion criteria. All patients carried a diagnosis of acute GVHD with the exception of Patient 4, who had suspected acute on chronic GVHD of the GI tract (Table 1). Retrospective chart review was performed in each case to understand pre-treatment GVHD therapies, post-treatment laboratory and clinical outcomes, and potential side effects for a period extending six weeks from the date of first administration. Two patients underwent HLA-matched related sibling donor SCT, one underwent SCT from an HLA-matched unrelated donor, one underwent an HLA-mismatched cord blood transplant, and two underwent haploidentical SCT from a related donor.

### 2.2. Methylprednisolone Therapy Administration 

The patient was placed supine on the angiography table, and the groin was prepped and draped in sterile fashion. Under ultrasound guidance, the left/right common femoral artery was accessed, and a 4Fr sheath was inserted, followed by heparin administration at 50 IU/kg. Selective cannulation of vessels was then performed using a co-axial system (base and microcatheter). For gastrointestinal (GI) disease, the superior mesenteric artery (SMA) and inferior mesenteric artery (IMA) were primarily cannulated. For those with upper GI involvement, the gastroduodenal artery (GDA) was also cannulated, and for those with lower GI involvement, bilateral internal iliac arteries (IIAs) were cannulated. For liver disease, the proper hepatic artery was selectively cannulated (or left and right hepatic arteries were individually cannulated if there was variant anatomy). Angiography was then performed to confirm location, after which methylprednisolone was infused over 10–15 min at each location (40–1000 mg, weight dependent, Table 1) for 10–15 min at each location. Steroid administration was then repeated on all affected disease locations. At the end of the case, the catheters and sheaths were removed from the patient, and hemostasis was achieved using manual compression for 10–15 min.

## 3. Results

### 3.1. Patient Characteristics

Six patients with intestinal and/or hepatic GVHD were treated with intraarterial methylprednisolone (Table 1). All had clinical grade 4 acute GVHD according to consensus grading by their primary SCT attending [17,18]. Three patients had GVHD of the GI tract, two had GVHD of the GI tract and liver, and one had GVHD of the liver without GI involvement. Of the five patients with GI involvement, two required frequent blood transfusions pre-procedure and were monitored in the intensive care unit setting (Patients 1 and 2); Patient 3 had stool output of roughly 5 L/day with occasional blood; Patients 3–5 had guaiac-positive stools but without critical GI tract involvement. All five patients were treated with systemic methylprednisolone (2 mg/kg except for Patient 1, who received a lower dose due to side effects) (Table 1). Of the four patients with liver involvement, three had biopsy-confirmed GVHD with typical features of interlobular bile duct injury (Patients 1, 5, and 6). 

### 3.2. Clinical Outcomes in GI GVHD

All patients successfully underwent arterial angiography with steroid delivery, and Patient 5 underwent three treatments with nine days separating each. Methylprednisolone was administered to the superior mesenteric artery (SMA), inferior mesenteric artery (IMA), internal iliac artery (IIA), and/or the gastroduodenal artery (GDA) (Table 1). Clinical response was observed within 24–48 h in all five patients. Patients 1 and 2 required ICU-level care pre-procedure, with Patient 2 requiring resuscitation with massive transfusion protocol (blood, platelets, and fresh frozen plasma) pre-procedure. Patients 1 and 2 had stabilized vital signs and were able to forgo further blood transfusion for at least one week post-procedure, although they continued to require platelet transfusions due to the profound cytopenia secondary to the GVHD and the multiple immunosuppressive agents administered. Patient 3 had a resolution of hematochezia 48 h after treatment, and at 96 h, the stool output decreased from 5 L/day to 2.5 L/day. While he had required daily blood transfusions pre-procedure, he also did not require further blood transfusion post-procedure. Patients 4 and 5 had resolution of guaiac-positive stools, while maintaining stable chronic anemia and thrombocytopenia. INR values for all patients remained stable at less than 1.4 during this period, with the exception of Patient 2, who experienced profound coagulopathy in the setting of massive GI hemorrhage. 

With regard to treatment durability, Patient 1 died two months after intraarterial steroid administration from respiratory and renal failure, although she did not have any further GI bleeding. Patient 2 developed a recurrent massive GI hemorrhage three weeks after treatment, but the family chose to forgo further medical care, and the patient died shortly thereafter. At the last follow-up, Patient 3 had a further decrease in stool output to 1.5 L/day two weeks after intraarterial steroid administration and started ruxolitinib with continued clinical response. Patient 4 had stools that remained guaiac-negative, although he continued to struggle with abdominal pain of unclear etiology despite extensive workup. Patient 5 was transitioned onto a combination of ruxolitinib, tacrolimus, and extracorporeal photopheresis (ECP); the patient was successfully weaned off systemic steroids. 

### 3.3. Clinical Outcomes in Hepatic GVHD

Four of the patients had acute hepatic GVHD: two had stage 4, and two had stage 2 disease, as graded by MAGIC criteria18. Of the patients with stage 4 involvement (1 and 6), Patient 1 had severe complex medical disease with respiratory and renal failure pre-procedure. The total bilirubin remained stably elevated following treatment. Patient 6 had isolated hepatic GVHD without GI involvement and experienced a rapid decline in total bilirubin following intraarterial methylprednisolone. His GVHD was ultimately managed with ECP and sirolimus, and steroids were successfully weaned. Patients 2 and 5, who had stage 2 liver disease, experienced a drop in total bilirubin levels shortly after intraarterial methylprednisolone therapy (within 72 h). However, Patient 5 had rebound cholestasis and was given two additional treatments, resulting in stably improved bilirubin. Patient 2′s bilirubin level remained stable until roughly 20 days after the procedure, when it increased again in the context of recurrent GI hemorrhage.

## 4. Discussion

Intraarterial methylprednisolone administration has shown promise as a potential salvage therapy in steroid-resistant acute GVHD in adults [9,10,11]. While one study included an unknown number of pediatric patients [19], this technique has not been reported in a pediatric cohort of patients outside selective case reports. Understanding the feasibility and efficacy of intraarterial steroid treatment for acute GVHD in children is important because this population suffers from a higher incidence of severe GVHD following SCT and is more likely to experience complications from systemic steroid administration [20,21,22]. The benefits of locoregional approaches to maximize target organ drug delivery must be weighed against the procedural risk in children, who are smaller than adults. For example, for arterial access, a smaller sheath size is favored with children also requiring heparinization to maintain vessel patency, which could be a procedural contraindication for some patients. The contrast load used in angiography is also a consideration, particularly for children with renal failure (such as Patient 1). Importantly, we observed no major adverse effects across seven treatments administered, which is consistent with angiography experiences in children with other serious medical conditions treated at large academic medical centers [14,15,16]. Most patients in this cohort had hypertension and hyperglycemia pre-procedurally due to longstanding systemic steroid administration; none of these conditions worsened following intraarterial steroid therapy. 

While our cohort is too small for the formal statistical analysis of the efficacy of intraarterial methylprednisolone for acute GVHD with GI involvement (including GI hemorrhage), the study showed a remarkable response from all patients with a response duration of roughly three weeks. For those with liver involvement, outcomes were more varied, with three of four patients responding. Whether renal failure is predictive of poor response remains unclear and should be further clarified in future studies. Patients in this cohort fell into two treatment categories: rescue therapy for patients already treated with an array of medications (e.g., Patient 1) and bridge therapy for patients treated with systemic steroids potentially alongside other agents but who started another medication around the time of intraarterial steroid administration that was expected to take days to weeks to have a clinical effect (e.g., Patient 3, who started ruxolitinib and vedolizumab after intraarterial steroids). All of the patients in this study had been previously treated with systemic methylprednisolone (most commonly 2 mg/kg daily); intraarterial doses ranged from 5–10 mg/kg in those with inflamed bowels to 20 mg/kg given to Patient 5 [8]. Additional studies will need to optimize the dosing of intraarterial methylprednisolone for GVHD, particularly in light of the immediate vs. delayed immunomodulatory effects of glucocorticoids, which are relevant for patients who receive high doses of steroid therapy in the setting of long-term systemic use [23]. In this study, the smallest patient weighed 37.6 kg. We previously reported on the use of a similar technique to deliver methylprednisolone intraarterially to the bowel of a 10-year-old patient with inflammatory bowel disease who weighed 24 kg, but future work will need to assess the feasibility and safety of this procedure more thoroughly in younger children [8]. In summary, we found that intraarterial methylprednisolone administration was feasible in pediatric patients with gastrointestinal and liver acute GVHD and shows promise as a potential salvage therapy for gastrointestinal bleeding and cholestasis. Other therapies could also be adopted using locoregional approaches; mesenchymal stem cell therapy is under investigation as a novel treatment for GVHD [24], as are monoclonal antibodies targeting CD25 and CD26, surface expression markers thought to play a central role in GVHD.

## Figures and Tables

**Table 1:** Clinical characteristics and outcomes of pediatric patients with GVHD treated with locoregional methylprednisolone.

Patient	Patient Background	Age	Sex	Weight (kg)	Pre-Treatment GVHD Medications	Procedure Indication(s)	Vessel(s) Accessed, (Methylprednisolone Dose), and Total Dose	Symptom Improvement	Sustained Response (>2 Weeks)	Treatment Effect(s)
1	AML s/p 2nd MSD SCT w/grade 4 acute GvHD: liver (stage 4), skin (stage 1), and GI (stage 4).	21	F	92.7	Methylprednisolone ECP Vedolizumab Ruxolitinib Sirolimus Infliximab Tacrolimus	Lower GI hemorrhage, diarrhea, and cholestasis	Bilateral IIA (40 mg each), IMA (50 mg), SMA (50 mg), proper hepatic artery (1000 mg). Total dose: 12.7 mg/kg.	Yes	Yes	Rapid resolution of GI bleeding and diarrhea
2	AML s/p 1st MUD SCT w/grade 4 acute GvHD: skin (stage 4) and GI (stage 4).	11	M	41.3	Methylprednisolone ECP Infliximab Begelomab Ruxolitinib	Lower GI hemorrhage and cholestasis	Bilateral IIA (40 mg each), IMA (60 mg), SMA (60 mg), proper hepatic artery (1000 mg). Total dose: 29.1 mg/kg.	Yes	Yes	Rapid resolution of GI bleeding
3	T-cell lymphoma s/p unrelated cord blood SCT w/grade 4 acute GvHD: GI (stage 4) and liver (stage 4)	12	M	44.5	Methylprednisolone Tacrolimus Basiliximab Mycophenolate	Diarrhea and lower GI bleeding	SMA (115 mg), IMA (115 mg), GDA (55 mg), left gastric artery (55 mg). Total dose: 7.6 mg/kg.	Yes	Yes	Rapid resolution of GI bleeding and diarrhea improved moderately
4	CML s/p 1st MSD SCT w/grade 4 acute on chronic GVHD: GI (stage 4) and skin (stage 3).	19	M	47.8	Methylprednisolone Tacrolimus Basiliximab Ruxolitinib	Diarrhea and guaiac positive stool	GDA (40 mg), SMA (60 mg), Ileocolic artery (20 mg), IMA (60 mg). Total dose: 3.8 mg/kg.	Yes	Yes	Improved diarrhea, resolution of GI bleeding, and transient improvement in abdominal pain
5	FSGS s/p 1st haploidentical SCT w/grade 4 acute GVHD: skin (stage 4) and liver (stage 2).	19	M	56.7	Methylprednisolone Basiliximab Begelomab ECP Cyclosporine	Guaiac positive stool and cholestasis	Left IIA (40 mg), IMA (60 mg), SMA (60 mg), LHA (500 mg), RHA (500 mg). Total dose: 20.5 mg/kg.	Yes	No	Required two additional treatments; guaiac stools resolved
6	B cell ALL s/p 1st haploidentical SCT w/grade 4 acute GVHD: liver (stage 4)	12	M	37.6	Methylprednisolone ECP Sirolimus	Cholestasis	RHA (500 mg), LHA (350 mg), accessory LHA (150 mg). Total dose: 26.6 mg/kg.	Yes	Yes	Improved cholestasis

AML: Acute myeloid leukemia; SCT: stem cell transplant; ECP: extracorporeal photopheresis; IIA: internal iliac artery; IMA: inferior mesenteric artery; SMA: superior mesenteric artery; GDA: gastroduodenal artery; RHA: right hepatic artery; LHA: left hepatic artery.

## Data Availability

The data from this study are not publicly available to protect patient privacy.
